# THE IMPACT OF THE COVID-19 PANDEMIC ON FAMILY CARE PARTNERS OF PERSONS WITH DEMENTIA IN ASSISTED LIVING FACILITIES

**DOI:** 10.1093/geroni/igad104.1769

**Published:** 2023-12-21

**Authors:** Inga Antonsdottir

**Affiliations:** Johns Hopkins University, Baltimore, Maryland, United States

## Abstract

**Background:**

The Covid-19 pandemic affected persons living with dementia and their family care-partners. Family care-partners are diverse, including spouses, children, and friends. Care-partners may live with the care recipient, or apart from them. Covid-19 policies and priorities isolated care-partners living at home, while banning others from supporting their loved ones in assisted living facilities (ALF). Purpose: The purpose of this study was to examine the impact the covid-19 pandemic had on family care-partners of persons living with dementia.

**Methods:**

This study used an online survey of 133 family care-partners (89 living in ALF, 44 living in the community).

**Results:**

Among family care-partners, 34.8% of those who lived in an ALF reported a severe change in routine compared to 38.6% of those living in the community. 78.7% of ALF care-partners reported that they were unable to visit their loved one in a care facility, compared to 31% of care-partners living in the community. 67.4% of ALF care-partners were separated from the person with dementia that they cared for, compared to 22.7% of care-partners living in the community. 70.5% of CPs in the community reported they had to spend a lot more time taking care of a family member with dementia compared to 13.5% of ALF CPs.

**Conclusion:**

Unique differences exist in how the pandemic impacted the lives of family care-partners of community-based or ALF-based persons living with dementia. Understanding these differences can help tailor policies and healthcare priorities to best support family care-partners living in community or ALF settings.

